# Diffusion tensor imaging for assessment of microstructural changes associate with treatment outcome at one-year after radiofrequency Rhizotomy in trigeminal neuralgia

**DOI:** 10.1186/s12883-019-1295-5

**Published:** 2019-04-12

**Authors:** Shu-Tian Chen, Jen-Tsung Yang, Hsu-Huei Weng, Hsueh-Lin Wang, Mei-Yu Yeh, Yuan-Hsiung Tsai

**Affiliations:** 1 Department of Diagnostic Radiology, Chang Gung Memorial Hospital Chiayi Branch, No.6 Chia-Pu Rd. West Sec., Chiayi County, Taiwan; 2 Department of Neurosurgery, Chang Gung Memorial Hospital Chiayi Branch, Chiayi, Taiwan; 3 Department of Biomedical Engineering and Environmental Sciences, National Tsing Hua University, Hsinchu, Taiwan

**Keywords:** Trigeminal neuralgia, Radiofrequency rhizotomy, Diffusion tensor imaging, Nerve volume, Treatment outcome

## Abstract

**Background:**

Trigeminal neuralgia (TN) is characterized by facial pain that may be sudden, intense, and recurrent. Neurosurgical interventions, such as radiofrequency rhizotomy, can relieve TN pain, but their mechanisms and effects are unknown. The aim of the present study was to investigate the microstructural tissue changes of the trigeminal nerve (TGN) in patients with TN after they underwent radiofrequency rhizotomy.

**Methods:**

Thirty-seven patients with TN were recruited, and diffusion tensor imaging was obtained before and two weeks after radiofrequency rhizotomy. By manually selecting the cisternal segment of the TGN, we measured the volume of the TGN, fractional anisotropy (FA), apparent diffusion coefficient (ADC), axial diffusivity (AD), and radial diffusivity (RD). The TGN volume and mean value of the DTI metrics of the post-rhizotomy lesion side were compared with those of the normal side and those of the pre-rhizotomy lesion side, and they were correlated to the post-rhizotomy visual analogue scale (VAS) pain scores after a one-year follow-up.

**Results:**

The alterations before and after rhizotomy showed a significantly increased TGN volume and FA, and a decreased ADC, AD, and RD. The post-rhizotomy lesion side showed a significantly decreased TGN volume, FA, and AD compared with the normal side; however, no significant difference in the ADC and RD were found between the groups. The TGN volume was significantly higher in the non-responders than in the responders (*P* = 0.016).

**Conclusion:**

Our results may reflect that the effects of radiofrequency rhizotomy in TN patients include axonal damage with perineural edema and that prolonged swelling associated with recurrence might be predicted by MRI images. Further studies are necessary to understand how DTI metrics can quantitatively represent the pathophysiology of TN and to examine the application of DTI in the treatment of TN.

## Background

Trigeminal neuralgia (TN) is a common cause of facial pain and is characterized by a recurrent sudden onset of electric shock-like pain that is localized to the sensory supply area of the trigeminal nerve (TGN). TN is typically induced by a normally non-painful mechanical irritation, and TN patients are usually pain-free between pain attacks [1]. The most common cause of TN is neurovascular compression of the TGN at the root entry zone [2], although the exact pathogenesis is still debated. Previous studies on the pathology of TN demonstrated demyelination of the TGN in patients with TN by ultrastructural and histological analyses [2–4]. The alteration of diffusion tensor imaging (DTI) metrics, including decreased fractional anisotropy (FA), increased radial diffusivity (RD), and no change in axial diffusivity (AD), could identify the same microstructural abnormality by non-invasive means [5–12].

Trigeminal neuralgia is treated by anticonvulsants, microvascular decompression, or minimally invasive percutaneous lesioning of the TGN, such as radiofrequency rhizotomy [13]. Radiofrequency rhizotomy was first used to treat chronic pain in 1974 [14], and Lopez BC et al. showed that percutaneous radiofrequency rhizotomy provides a high satisfaction with complete pain relief and low side effects. Among the various interventional pain therapies, radiofrequency rhizotomy provides the highest initial pain free experience; however, 15–20% of patients experience recurrent TN within 12 months [15].

Several studies have found abnormal DTI metrics and volume changes at trigeminal nerve in patients with TN [6, 9, 16–19]. Liu et al. reported that the FA reduction is correlated with visual analogue scale (VAS) [9], and DeSouza et al. demonstrated DTI metrics correlated with pain scores following treatment [16], which suggests that DTI metrics could be an imaging biomarker for monitoring clinical severity and treatment outcomes. By MRI volumetry, the preoperative volume of affected trigeminal nerve was significant reduced at cistern segment compared to the unaffected side in patients with TN [6, 17, 18]. Leal et al. [20] further suggested that the volume variance is significantly correlated with the severity of the compression; there is a smaller TGN volume in Grade 3 (indentation) than in Grade 1 (contact). However, it is not clear whether volume variance or DTI metrics can help predict long-term outcomes after intervention. The aim of this study was to investigate the microstructural tissue changes before and after radiofrequency rhizotomy of the TGN in patients with TN by multiple DTI metrics (FA, AD, and RD) and the nerve volumetric change and to determine whether recurrence can be predicted with DTI metrics obtained at the initial post-rhizotomy evaluation.

## Methods

### Participants

**Fig. 1:**
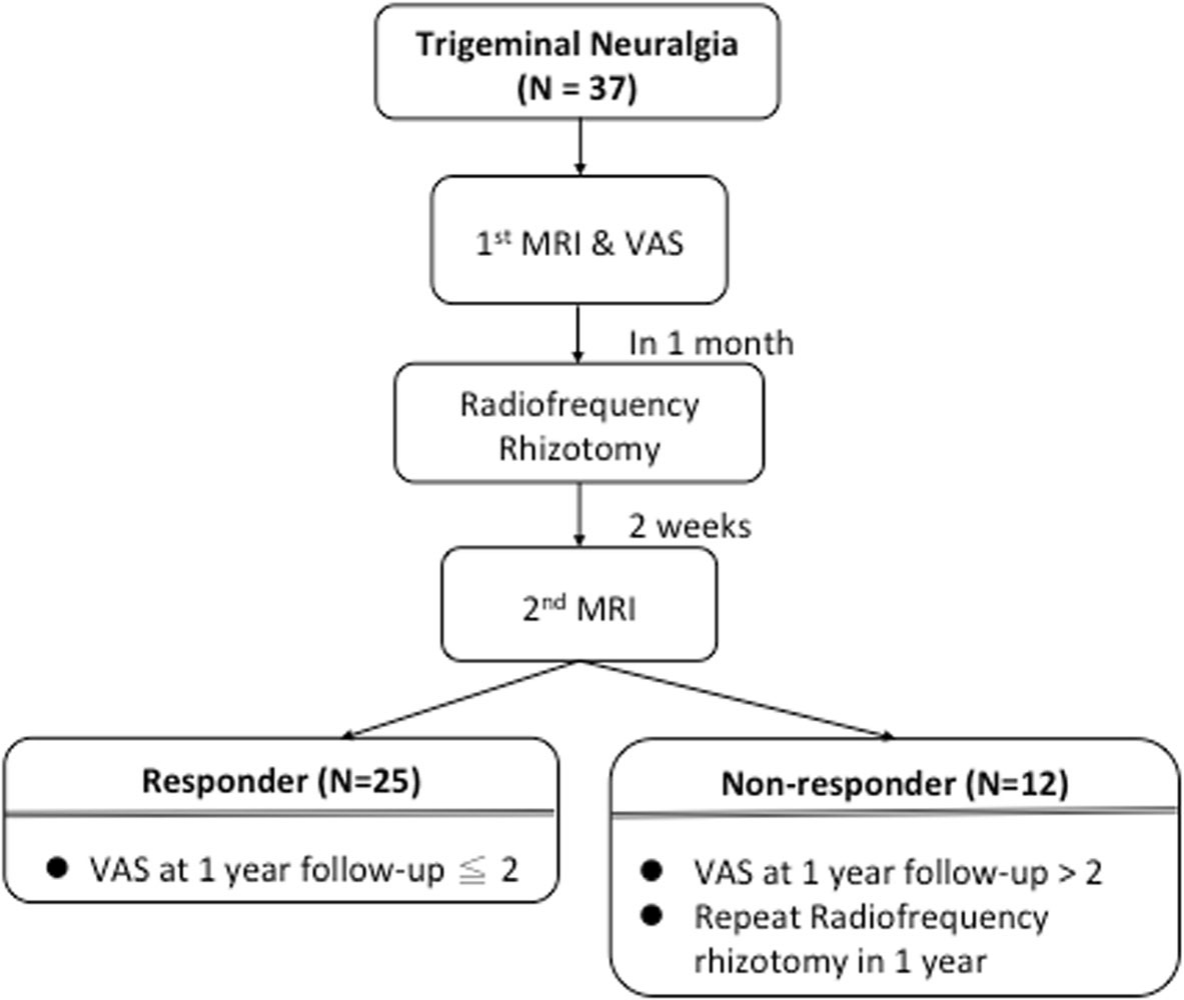
A flowchart of the patient selection and study workflow

Thirty-seven patients with TN were prospectively enrolled in this study. All of the patients were diagnosed as having TN according to the criteria of the International Headache Society for TN [21]. All of the patients underwent first-time MRI and received radiofrequency rhizotomy less than 1 month between the first-time MRI and the clinical evaluation. Post-interventional MRI was performed 2 weeks after the radiofrequency rhizotomy. Additionally, the VAS pain scores were assessed twice, once before the rhizotomy (pre-rhizotomy VAS) and 1 year after the rhizotomy (post-rhizotomy VAS). Specifically, post-rhizotomy VAS scores of 0, 1, and 2 are interpreted as responders, and a post-rhizotomy VAS score of more than 2 and receiving secondary rhizotomy within 1 year are interpreted as non-responders (Fig. 1). Written informed consent was obtained from each participant, and the institutional review board of Chang Gung Memorial Hospital at Chiayi approved this study.

### MRI acquisition and processing

All of the data were collected with a 3 Tesla Siemens Verio MRI system (Siemens Medical System, Erlangen, Germany) using a 32-channel head coil. The DTI sequences were obtained using a readout-segmented echoplanar imaging (RS-EPI) sequence (Syngo RESOLVE; Siemens Medical System) with the following parameters: matrix size = 110 × 110; FOV = 220 mm; section thickness = 2 mm; readout segments = 5; slice = 20 without a gap; b value = 0 and 1000 s/mm^2^; diffusion directions = 30; TR = 2800 ms; TE1/TE2 = 70 ms/95 ms; spatial resolution = 2 mm × 2 mm × 2 mm; echo spacing = 0.32 ms; echo reading time = 7.04 ms; and acquisition time: 8 min and 51 s. 3D MP-RAGE anatomical images were obtained using a gradient echo sequence with the following parameters: TR = 1900 ms; TE = 2.98 ms; FOV = 230 mm; matrix = 220 × 256; slice number: 160; spatial resolution of 0.9 mm × 0.9 mm × 0.9 mm; and acquisition time: 5 min and 59 s. DSI Studio software package utilities (http://dsi-studio.labsolver.org/) were used for the post-processing of the DTI data. The methods used for processing the DTI data have been previously reported [10]. Briefly, the DTI maps were co-registered to the 3D MP-RAGE anatomical images in the axial plane. Then, the regions of interest (ROIs) were placed onto the co-registered image and at the slice, which has the largest number of voxels at the cistern segment of the TGN. All of the imaging voxels covering the cisternal segment of the TGN were manually selected on the DTI images by two independent neuroradiologists (YH Tsai and HH Weng) who were blinded to the patient data, including the side of pain and surgical outcome. The trigeminal cistern segment ROI was 7 voxels in size. The average DTI metrics of all of the voxels within the ROI, including the ADC, FA, AD, and RD, were then separately calculated by the two observers. The volume of the cisternal segment of the TGN was manually measured on the 3D MP-RAGE anatomical images using ImageJ software (https://imagej.nih.gov/ij/).

### Radiofrequency rhizotomy

Percutaneous radiofrequency rhizotomy was performed by an experienced neurosurgeon (JT Yang). The rhizotomy needle was inserted under CT guidance, and the precise location was confirmed by three-dimensional image reconstruction using 1.25 mm-thick slices (Advantage Workstation 4.0, GE Medical Systems, WI, U.S.A.). The subsequent location and lesioning were determined by the reproduction of paresthesia upon stimulation covering the distribution of a specific division of the TGN. The lesion at the Gasserian ganglion was made by radiofrequency thermocoagulation (Radionics, Inc. Burlington, MA, USA) at 65 °C for 100 s and then at 70 °C for another 100 s [22, 23].

### Statistical analysis

All of the DTI metrics, including the ADC, FA, AD, and RD, were tested for normality of distribution using the Kolmogorov-Smirnov test. The volumes and values of the DTI metrics of the post-rhizotomy lesion side of the TGN were compared to those of the normal side and to those of the pre-rhizotomy lesion side by using a paired sample t-test. In the analysis of the prognosis of the patient, an independent sample t-test was used to compare the mean FA, ADC, AD, and RD between the responders and non-responders. A comparison between the baseline characteristics of the responders and the non-responders was assessed by using the Mann-Whitney U test and Fisher exact test. Multiple comparisons were statistically corrected with Bonferroni procedure (*p* < 0.05/7). For statistical analysis, we used the calculated mean values from the two observers. Inter-observer agreement was examined using the intraclass correlation coefficient (ICC). All of the statistical calculations were performed with SPSS V.18 software (SPSS, Chicago, IL).

## Results

### Baseline characteristics

The baseline characteristics of the participants are summarized in Table 1. A total of 37 patients were included, 13 males and 24 females, aged 43–87 years (mean 59.8 years). The left side was affected in 11 of the patients, while the right side was affected in 26 of the patients. The mean disease duration was 92.7 ± 89.4 months.

**Table 1:** Summary of the patient characteristics

Characteristic	Mean (SD) or n (percentage)
Total number of patients	37
Age, yr.	59.8 (7.6)
Male gender	13 (35.1%)
Left side	11 (29.7%)
Duration, mo.	92.7 (89.4)
VAS	
Pre-Radiofrequency rhizotomy	9.2 (0.9)
Post-Radiofrequency rhizotomy	2.2 (3.2)

### DTI metrics of lesion side TGN: a comparison between pre-rhizotomy and post-rhizotomy

**Fig. 2:**
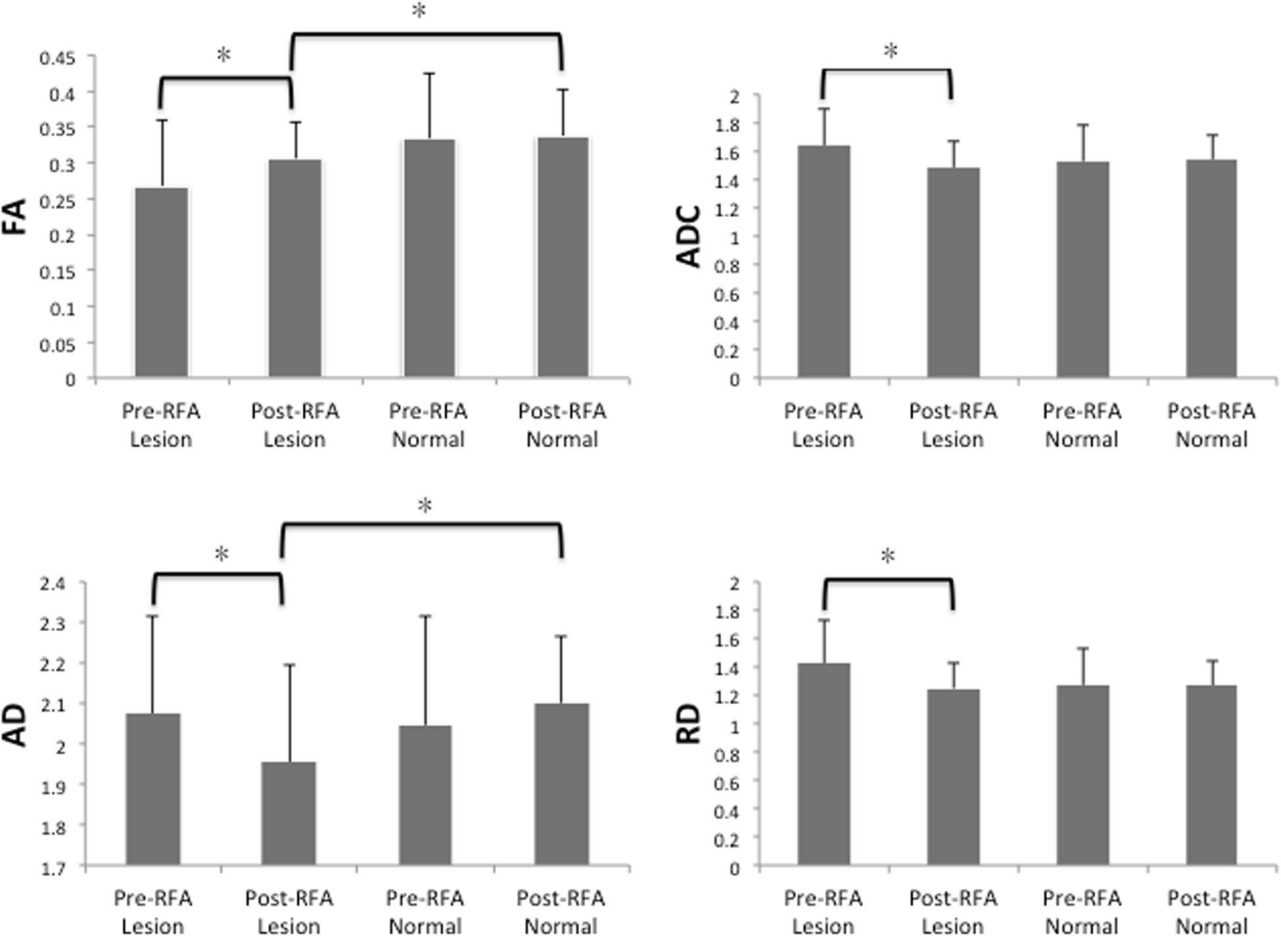
Bar charts of the DTI metrics in the lesion and normal sides and of the ablated and untreated sides after radiofrequency rhizotomy (RFA). A significant increase in the FA and decreases in the ADC, AD and RD were noted in a lesion undergoing RFA. (FA: fraction anisotropy; ADC: apparent diffusion coefficient; AD: axial diffusivity; RD: radial diffusivity)

The ICC showed a good inter-observer reliability for the measurement of the pre-rhizotomy FA of the affected TGN (average measure of the ICC = 0.898). The differences in the pre-rhizotomy and post-rhizotomy DTI metrics of the lesion side are shown in Table 2 and Fig. 2. The post-rhizotomy volume of the TGN (56.4 ± 25.0 mm^3^) was significantly increased compared to the pre-rhizotomy volume of the TGN (48.6 ± 18.7) (*P* = 0.014). The post-rhizotomy FA (0.306 ± 0.051) was greater than the pre-rhizotomy FA (0.268 ± 0.093) (*P* = 0.015) but not significant after multiple comparison correction. The ADC, AD, and RD were lower at post-rhizotomy (1.484 ± 0.190 × 10^− 3^ mm^2^/s, 1.953 ± 0.244 × 10^− 3^ mm^2^/s, and 1.249 ± 0.177 × 10^− 3^ mm^2^/s, respectively) than at pre-rhizotomy (1.640 ± 0.261 × 10^− 3^ mm^2^/s, 2.075 ± 0.242 × 10^− 3^ mm^2^/s, and 1.423 ± 0.299 × 10^− 3^ mm^2^/s, respectively) (*P* = 0.001, 0.016, and 0.001, respectively). The difference of AD did not reach statistically significant after multiple comparison correction.

**Table 2:** Summary of the differences between the pre-radio frequency rhizotomy and post-radiofrequency rhizotomy DTI metrics of the lesion side (*N* = 37)

	Pre-rhizotomy (SD)	Post-rhizotomy (SD)	P value
Volume (mm^3^)	48.6 (18.7)	56.4 (25.0)	0.014*
Fractional anisotropy	0.268 (0.093)	0.306 (0.051)	0.015*
Apparent diffusion coefficient (*10^−3^)	1.640 (0.261)	1.484 (0.190)	0.001*
Axial diffusivity (*10^−3^)	2.075 (0.242)	1.953 (0.244)	0.016*
Radial diffusivity (*10^−3^)	1.423 (0.299)	1.249 (0.177)	0.001*

*P < 0.05 was considered to indicate a significant difference

### Post-rhizotomy DTI metrics of the TGN: a comparison between the lesion side and contralateral side

**Fig. 3:**
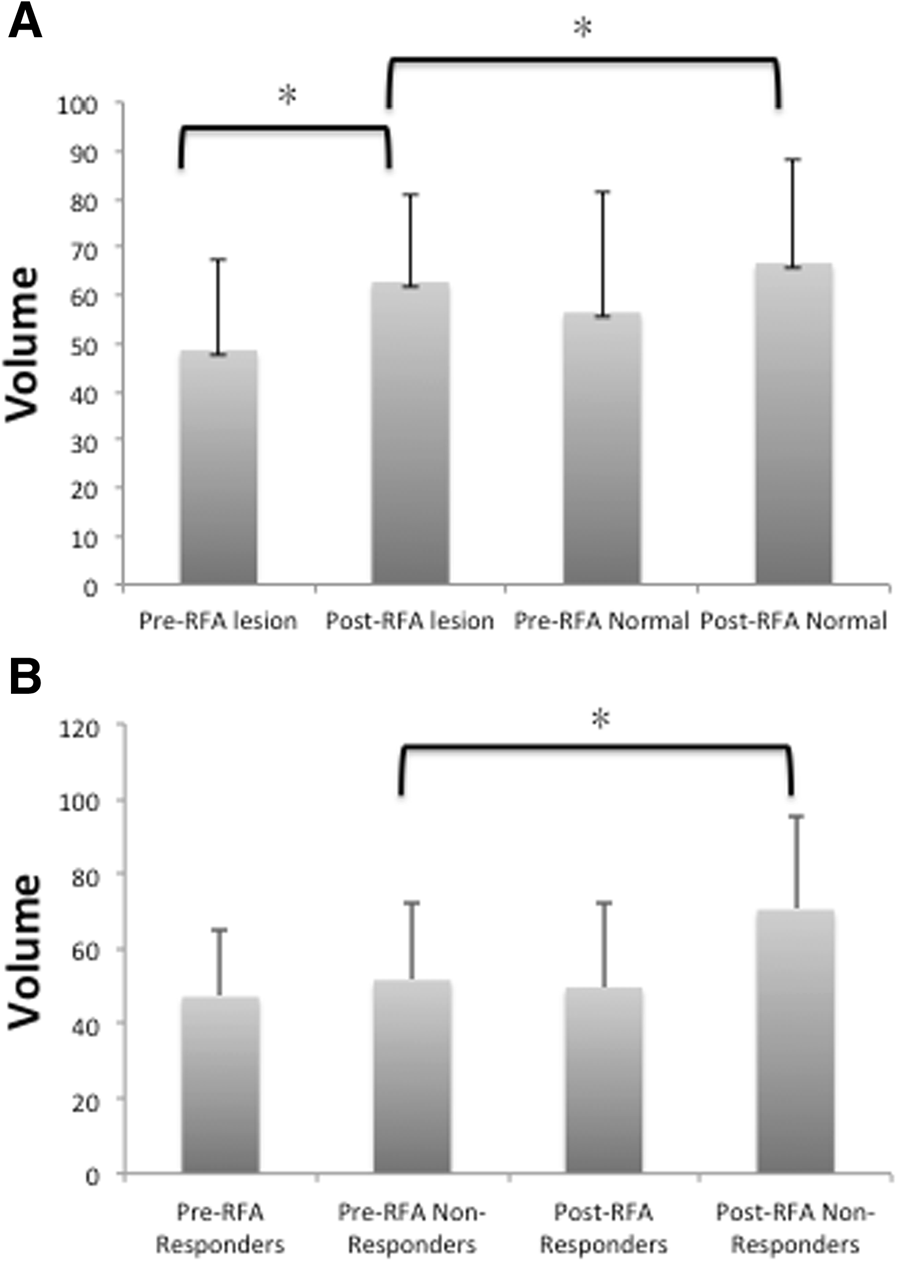
Bar charts of the volumes (**a**) in the lesion and normal sides and in the ablated and untreated sides after radiofrequency rhizotomy (RFA) (**b**) in the ablated side of the responders and non-responders. **a** A significantly increased TN volume in the lesion side after RFA is shown. **b** A significantly increased volume in the ablated side is shown in the non-responders after RFA, but no change is shown in the responders after RFA

The differences in the DTI metrics between the lesion side and contralateral side after the rhizotomy are shown in Table 3. The volume of the TGN of the lesion side (56.4 ± 25.0) was significantly smaller than that of the unaffected side (66.6 ± 21.8) (*P* = .005) (Fig. 3a). The FA and AD of the affected side were significantly lower than those of the unaffected side (*P* = 0.012 and 0.001, respectively). However, after multiple comparison correction, FA was not statistically significant. There were no statistically significant differences between the affected and unaffected sides of the patients for the ADC and the RD (*P* = 0.075 and 0.640, respectively) (Fig. 2).

**Table 3:** Summary of the differences in the DTI metrics between the lesion side and contralateral side of the trigeminal nerve after radiofrequency rhizotomy (N = 37)

	Lesion Mean (SD)	Normal Mean (SD)	P value
Volume (mm^3^)	56.4 (25.0)	66.6 (21.8)	0.005*
Fractional anisotropy	0.306 (0.051)	0.338 (0.063)	0.012*
Apparent diffusion coefficient (*10^−3^)	1.484 (0.190)	1.544 (0.164)	0.075
Axial diffusivity (*10^−3^)	1.953 (0.244)	2.101 (0.163)	0.001*
Radial diffusivity (*10^−3^)	1.249 (0.177)	1.265 (0.177)	0.640

*P* < 0.05 was considered to indicate a significant difference

### Therapeutic outcomes

The baseline characteristics of the responders and non-responders are shown in Table 4. There were no significant differences in the age, sex, lesion side, disease duration, and pre-rhizotomy VAS score between the responders and non-responders (*P* = 0.618, *P* = 0.874, *P* = 0.228, *P* = 0.616, and *P* = 0.059, respectively). The TGN volume of the pre-rhizotomy lesion side and DTI metrics also showed no significant differences between groups. After the rhizotomy, the volume of the TGN of the lesion side was significantly higher in the non-responders (70.4 ± 24.9 mm^3^) than in the responders (49.7 ± 22.6) (*P* = 0.016) (Fig. 3b), yet no significant differences in the post-RFA FA, ADC, AD and RD (Table 4).

**Table 4:** Summary of the characteristics of the responders and the non-responders

	Responders (*n* = 25)	Non-responders (*n* = 12)	*P* value
Age, yr.	59.4 (8.2)	60.8 (6.1)	0.618
Male	9 (36.0%)	4 (33.3%)	0.874
Left side	9 (36.0%)	2 (16.7%)	0.228
Duration, mo.	97.9 (91.5)	81.8 (87.7)	0.616
Pre-rhizotomy VAS	9.5 (0.7)	8.7 (1.2)	0.059
Pre-rhizotomy lesion side			
Volume (mm^3^)	47.2 (18.0)	51.7 (20.6)	0.496
Fractional anisotropy	0.277 (0.104)	0.249 (0.064)	0.402
Apparent diffusion coefficient (*10^−3^)	1.617 (0.261)	1.690 (0.268)	0.434
Axial diffusivity (*10^−3^)	2.058 (0.241)	2.110 (0.251)	0.548
Radial diffusivity (*10^−3^)	1.396 (0.306)	1.480 (0.288)	0.434
Post-rhizotomy lesion side			
Volume (mm^3^)	49.7 (22.6)	70.4 (24.9)	0.016*
Fractional anisotropy	0.302 (0.043)	0.315 (0.066)	0.475
Apparent diffusion coefficient (*10^−3^)	1.470 (0.169)	1.513 (0.235)	0.527
Axial diffusivity (*10^−3^)	1.930 (0.216)	2.000 (0.300)	0.424
Radial diffusivity (*10^−3^)	1.240 (0.155)	1.269 (0.221)	0.641

Note – The values are the mean (standard deviation) or number (percentage)

*P < 0.05 was considered to indicate a significant difference

## Discussion

This paper is an extension of our previous study [10] -- further explorations of longitudinal microstructural changes of trigeminal nerve after radiofrequency rhizotomy using MRI. Besides, we try to identify prognostic imaging biomarker by MRI that performed 2 weeks after rhizotomy. As mentioned in the previous study, forty-seven patients with TN were prospectively enrolled into this study in the beginning, while four patients who had history of TN on the contralateral side were excluded. Among the 43 patients with unilateral TN, 37 received radiofrequency rhizotomy after MRI. The result of the previous study showed that there was no correlation between pre-rhizotomy DTI metrics, volume and the effective VAS score reduction at one-month follow up [10].

**Fig. 4:**
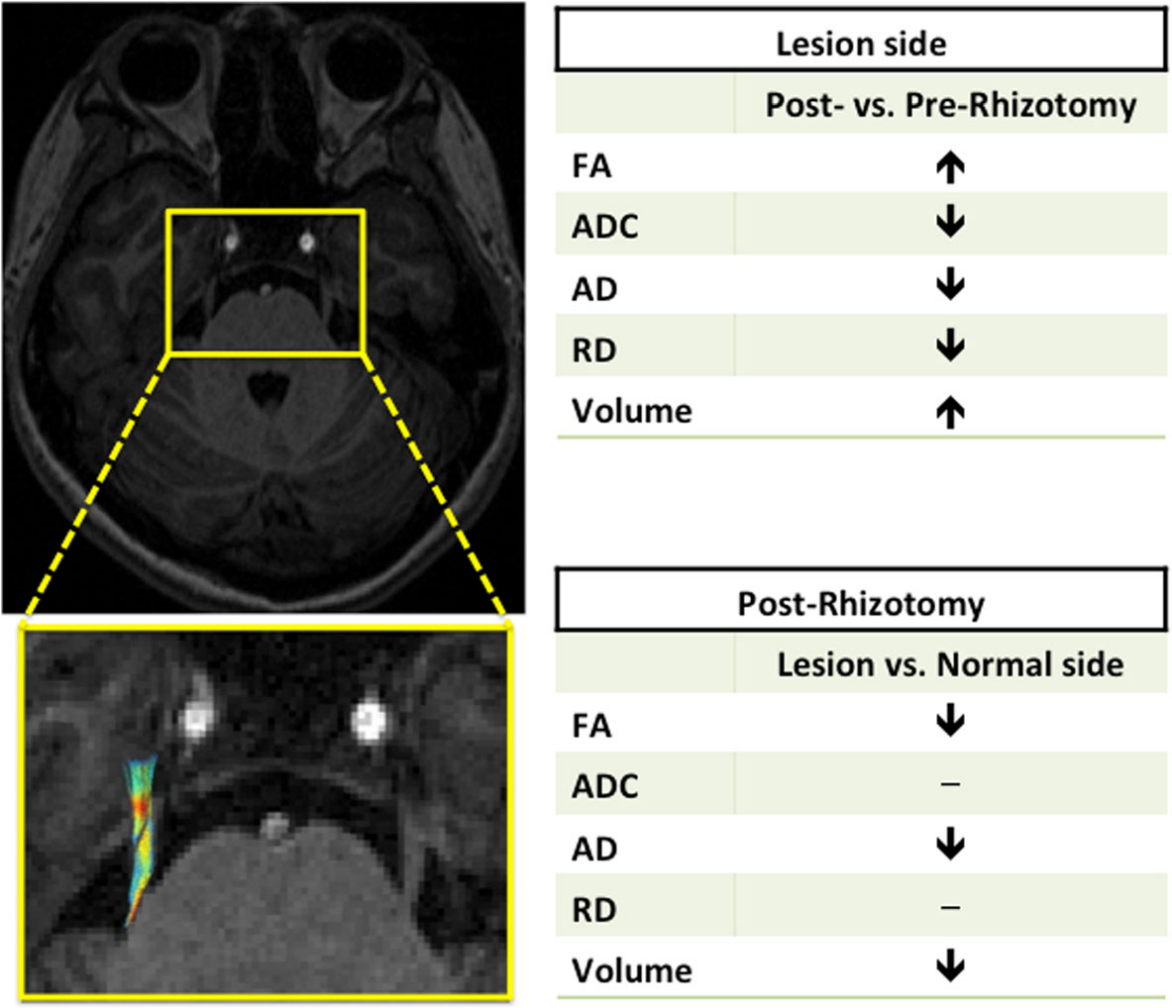
A summary of changes of the volume and diffusion tensor metrics of the trigeminal nerve in a patient with trigeminal neuralgia is shown. Upper table: a comparison between the TN of the lesion side before and after RFA. Lower table: a comparison between the TN of the lesion and normal sides after RFA. (FA: fractional anisotropy; ADC: apparent diffusion coefficient; AD: axial diffusivity; RD: radial diffusivity; RFA: radiofrequency rhizotomy)

In this study, we demonstrated that patients with trigeminal neuralgia who received radiofrequency rhizotomy may have had axonal injury with perineural edema at the cisternal segment of the TGN after the intervention. These microstructural abnormalities are characterized by a higher FA and lower ADC, AD, and RD in the post-rhizotomy lesion side compared with the pre-rhizotomy lesion side and also by a decreased FA and AD compared with the normal side. The TGN volume of the lesion side increased after radiofrequency rhizotomy, but the volume is still smaller than that of the unaffected side (Fig. 4). We also observed a significantly higher TGN volume of the post-rhizotomy lesion side in the non-responders compared to that of the responders, and there was no significant difference in the volume before the radiofrequency rhizotomy between the groups (*P* = 0.496).

Diffusion tensor imaging is based on the diffusion of free water protons along multiple directions in space, which enable the assessment of tissue architecture and microdynamics in vivo [24]. FA and ADC are parameters that are commonly used that represent a simplified description of water diffusion. Directional diffusivity metrics including axial and radial diffusivity (AD and RD) give additional evaluations of diffusivity parallel and perpendicular to fiber orientation, respectively, and are hypothesized to have a more specific differentiation of axonal integrity, demyelination, or edema [25, 26] as diffusion is particularly sensitive to changes in the architecture of cellular membrane under certain pathological conditions [12].

The histopathological changes of trigeminal nerve after radiofrequency lesioning are still debated. Previous studies assumed that radiofrequency rhizotomy treatment of TN is based on the fact that the Aδ and C fibers are more sensitive to thermocoagulation than the Aα and β fibers [27, 28]. Therefore, the irreversible damage to small, unmyelinated pain fibers blocks pain sensation without sensory and motor nerve damage when the temperature is from 55 °C to 70 °C [29]. However, recent research has shown that TN results from microstructural changes of trigeminal afferent neurons in the trigeminal root or ganglion and that the injury renders hyperexcitable axons [30], and pulsed radiofrequency damaged the trigger point which was mediated by peripheral low threshold myelinated Aβ fibers [31]. On the contrary, Choi et al. found the neurodestructive effect was severely and non-selectively degenerated and stunted myelinated axons, swelling and absence of mitochondria, complete destruction of collagen and elastin structure [32]. Our results of an increased volume and greater FA coupled with a lower ADC, AD, and RD are indicative of intracellular edema [33], neuroinflammation, and axonal alterations [34] at the cisternal segment in the TGN after radiofrequency rhizotomy. In addition, compared with the normal side, the affected side showing decreased FA and AD but no significant difference in the RD, which may indicate that there is axonal damage after radiofrequency rhizotomy. Axonal injury caused by rhizotomy may damage cell membrane structure and mitochondria causing increase in cell infiltration, which could potentially reduce extracellular fluid and overall diffusion [35]. Extracellular water diffuses into the cell interior, resulting in cell swelling and an increase in the TGN volume after rhizotomy, which is consistent with our findings. Our DTI and volume findings may support the non-selective effect of radiofrequency rhizotomy under aforementioned cellular mechanism. The post-rhizotomy pathologic findings include massive edema at 2 days after rhizotomy that progressed to Wallerian degeneration at 7–10 ± 14 days [36], which may give an explanation for ablation at the Gasserian ganglion causing tissue abnormalities at the root entry zone and pre-ganglion segment. Our results showed an increased TN volume at the time of 2 weeks post-rhizotomy, which probably indicated that the nerve is still edematous and that 2 weeks is too short of a time to cause volume loss.

Structural changes in the trigeminal nerve leading to volume loss have been well-documented. Leal et al. and Duan et al. attributed this volumetric change to atrophy and documented that the more severe atrophy of the TGN has a better clinical improvement following the surgical decompression of the nerve [20, 37]. However, it is not clear whether the volumetric change is entirely due to vessel compression or irreversible structural change. Furthermore, the correlation between the volume and outcome in treatments other than decompression surgery is not clear. We examined the effectiveness of radiofrequency rhizotomy at the time of one-year follow-up and how it impacts the cistern segment of the TGN by measuring the TGN volume and DTI metrics. Our results indicated that recurrence was associated with a significantly higher TGN volume without accompanying changes in the DTI metrics. Interestingly, there was no significant difference in the pretreatment baseline characteristics of the responders and non-responders, and there was no significant difference in TGN volume of the responders before and after rhizotomy (*P* = 0.496). The non-responders had a significantly increased TGN volume 2 weeks after the radiofrequency rhizotomy compared to before the rhizotomy (*P* = 0.016). These findings may indicate that prolonged cell swelling/inflammatory changes may be associated with recurrence. Additionally, an inadequate needle position during RFA may be the reason for recurrence, which causes a thermal effect mainly at the perineural tissue instead of at the nerve itself, thus having less of an effect of axonal damage to the TGN. Further study is indicated to support the current observation that the volume changes after RFA can be an imaging biomarker to predict recurrence.

There are several limitations to our study. First, the partial volume effect, especially from imaging voxels with a cerebrospinal fluid (CSF) signal, might lead to errors in the DTI measurement. In this study, we co-registered the DTI images to MPRAGE and selected the imaging voxels in the axial slice containing the most voxels of the TGN. Each voxel can be checked simultaneously in both the DTI and MPRAGE images to make sure that the voxel is within the TGN, and the procedure was double-checked by two observers, which produced a good ICC (0.898). Other limitations include that the study population was small and that the disease duration differed between the patients, which may cause different degrees of microstructural changes and treatment benefits. However, we found no correlation between the disease duration and DTI values.

## Conclusions

Our results may reflect that the effects of radiofrequency rhizotomy in TN patients include axonal damage with perineural edema and that prolonged swelling associated with recurrence might be predicted by MRI images. Further studies are necessary to understand how DTI metrics can quantitatively represent the pathophysiology of TN and to examine the application of DTI in the treatment of TN.

## Abbreviations

AD: Axial Diffusivity
ADC: Apparent Diffusion Coefficient
DTI: Diffusion Tensor Imaging
FA: Fractional Anisotropy
RD: Radial Diffusivity
TGN: Trigeminal Nerve
TN: Trigeminal Neuralgia
